# Necrotizing Fasciitis of the Breast: Case Report with Literature Review

**DOI:** 10.1155/2018/1370680

**Published:** 2018-10-23

**Authors:** Basem ALShareef, Nourah ALSaleh

**Affiliations:** Department of General Surgery, Al-Noor Specialist Hospital, Makkah, Saudi Arabia

## Abstract

Necrotizing fasciitis is a life-threatening aggressive soft tissue infection which usually affects the extremities, abdominal wall, or perineum. Breasts are rarely affected, with most cases presenting after trauma or surgical intervention. It may be misdiagnosed as abscess or cellulitis, leading to treatment delays. Here, we report a case of necrotizing fasciitis affecting both breasts in a 60-year-old female. Treatment included core biopsy managed with intravenous antibiotic and surgical debridement followed by a simple mastectomy. Currently, the patient is disease-free with a completely healed wound.

## 1. Introduction

Necrotizing fasciitis (NF) is one of the most severe and aggressive forms of soft tissue infections and is considered a life-threatening condition. It is characterized by spreading necrosis of subcutaneous tissue and fascia. It commonly affects the extremities, abdominal wall, or perineum. It rarely affects the breasts, and only a few cases have been reported, with most cases presenting after trauma or surgical intervention [1, 3, 4]. NF of the breast may be misdiagnosed for an abscess or cellulitis, and this can lead to treatment delays [4, 5].

## 2. Case Report

A 60-year-old postmenopausal African woman presented to the emergency department with a 6-month history of progressive bilateral breast pain and mass associated with itchiness. There was no history of fever, chills, discharge, or trauma and no previous breast surgery. Family history was negative for breast cancer. The patient had a history of diabetes mellitus, hypertension, and cardiomyopathy.

### 2.1. Physical Examination

On presentation, the patient was alert and oriented, with a temperature of 37°C, a pulse of 110/min, and blood pressure of 110/70 mmHg. Breast examination revealed a bilateral 7.5∗6 cm hard, fixed mass in the periareolar area with erythema and peau d'orange without discharges or palpable axillary lymph node. The rest of the examination was within normal.

**Figure 1:**
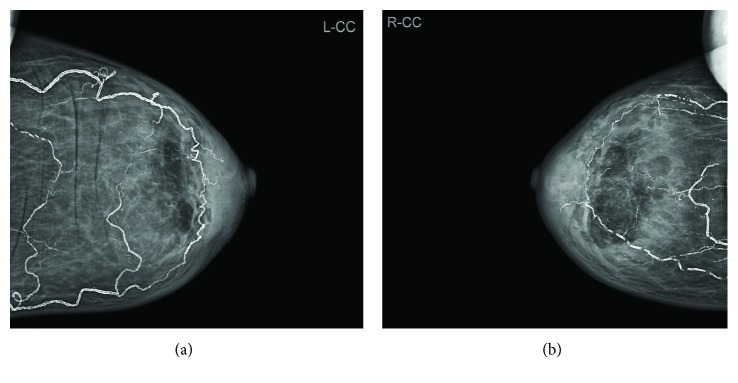
A mammogram study revealed left breast (a) and right breast (b) diffused skin thickening edematous parenchyma with vascular calcification.

A mammogram revealed bilateral diffused skin thickening edematous parenchyma with vascular calcification (Figure 1(a)) and 1.4∗0.8 cm hypoechoic lobulated irregular mass at the right breast (BIRADS 3) (Figure 1(b)). Bilateral core biopsies from both masses were taken.

**Figure 2:**
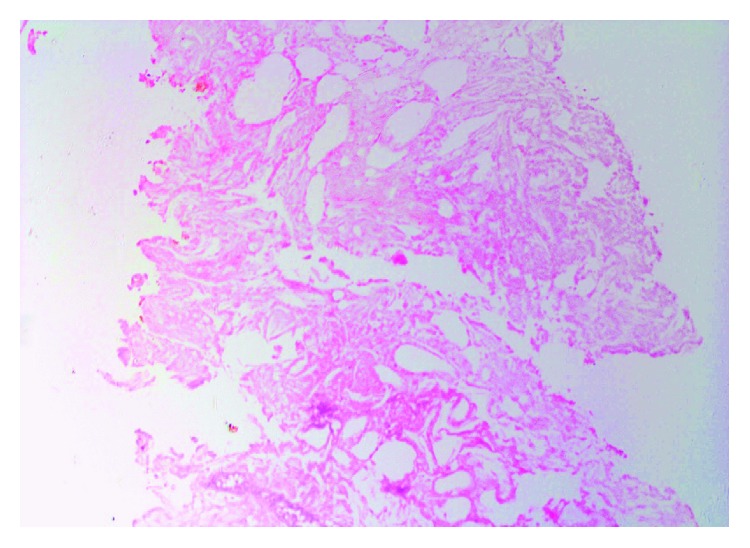
Core biopsy (×40, H&E stain) showing necrotic acutely inflamed fibrofatty tissue.

The histopathology result showed necrotic acutely inflamed fibrofatty tissue (Figure 2).

On follow-up, i.e., one week later, the patient presented with bilateral malodorous breast discharge at the biopsy site. On physical examination, both RT and LT breasts showed necrotic tissue with pus discharge and no crepitus and with palpable apical axillary lymph nodes.

Her laboratory results revealed leukocytes of 10.85∗10 mg/dL and elevated glucose of 148 mg/dL. She started on intravenous ceftriaxone and was taken to the operating theater for bilateral debridement and incisional biopsy as inflammatory breast cancer was suspected. Microscopic examination of specimens showed necrotic fibrofatty mammary tissue and foci of chronic inflammation. Two weeks later, the patient continued to have a nonhealing ulcer with foul-smelling discharge and expanding necrotic tissue. NF was suspected and the patient underwent bilateral simple mastectomy with primary wound closure by a stapler.

**Figure 3:**
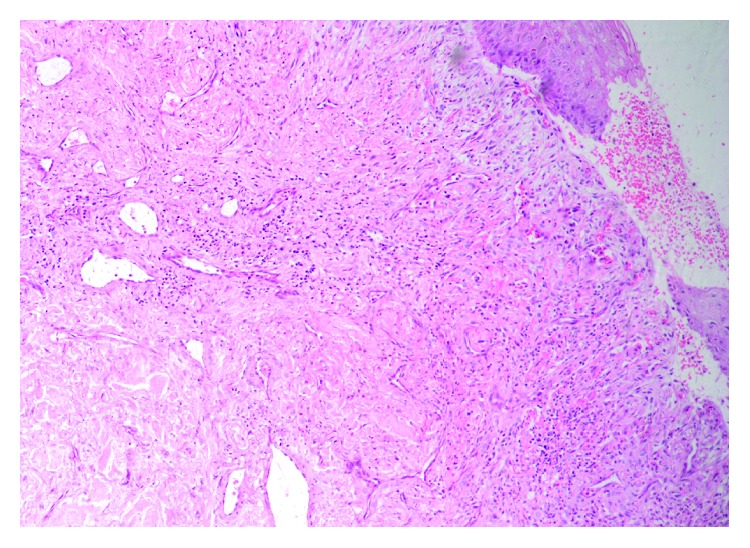
Core biopsy (×40, H&E stain) showing necrotizing inflammation lying around some scattered atrophic breast ducts with adjacent involved fat.

The histopathological examination of the specimens revealed an extensive cutaneous necrosis involving the epidermis, dermis, and subcutaneous fat with thrombus and necrosis of blood vessels (Figure 3) constant with necrotizing fasciitis. Postoperatively, she had an uneventful recovery and was discharged home after 3 days. Follow-up visits were arranged, and the patient was found to be completely healthy with a well-healed wound.

## 3. Discussion

Necrotizing fasciitis is a life-threatening, rapidly progressive infection [1] characterized by widespread necrosis of the subcutaneous tissue and fascia, with associated systemic toxicity and extension along fascial planes [2, 3]. Although NF can occur anywhere in the body, it commonly affects the extremities, followed by the trunk and perineum; only a few cases of NF in the breast have been reported, with the first reported case by Konil et al., Yaji et al., Fayman et al., Ward et al., and Shah et al. [1–5]. Literature reveals that necrotizing fasciitis of the breast is commonly misdiagnosed as cellulitis, mastitis, abscess, or inflammatory breast cancer as in our case [2, 4]. Predisposing risk factors include diabetes mellitus, peripheral vascular disease, alcoholic liver disease, immunosuppression, surgical wounds, and skin biopsies [1, 3–6]. Our reported patient had breasts' necrotizing fasciitis after core biopsies for bilateral breasts' mass, similarly reported by Lee et al. in 2015 [6] and Flandrin et al. in 2009 [7].

There are two bacterial forms of necrotizing fasciitis: type I necrotizing fasciitis is a mixed infection caused by aerobic and anaerobic bacteria and type II necrotizing fasciitis is generally monomicrobial and is typically caused by group A *Streptococcus* or other beta-hemolytic streptococci either alone or in combination with other pathogens [1, 4, 6, 7]. In our case, all cultures were negative due to antibiotic use. Many authors recommend that early debridement and appropriate antibiotic coverage significantly reduce both morbidity and mortality [2–4, 7] while mastectomy has been reported to be the main treatment for the majority of cases in the published literature [3, 4, 7] (Table 1). Konil et al., Yaji et al., Fayman et al., Ward et al., and Shah et al. suggested a six-point management plan for the treatment of such an infection including (1) early surgical referral, (2) resuscitation and antibiotic coverage, (3) diagnostic incision, (4) radical “pseudotumour” excision, (5) reexploration of the wound 24 hours later, and (6) delayed skin closure several months after recovery [1, 5]. In our case, we decided to treat the patient with bilateral simple mastectomy along with intravenous ceftriaxone as she presented with a large necrotic mass in comparison with her breast size.

## 4. Conclusions

Due to the rarity of necrotizing fasciitis of the breast, it may be misdiagnosed in the first presentation; however, if the patient has the mentioned risk factors along with the clinical presentation, necrotizing fasciitis should be considered as a differential. Although it is a rapidly progressive, life-threatening disease, early recognition and surgical intervention along with broad-spectrum antibiotic can greatly reduce morbidity and mortality. Histological examination of the tissue is important in confirming the diagnosis and ruling out cancer.

## Figures and Tables

**Table 1:** Existing case reports of NF in breast and management.

Author	Year	Patient age	Treatment
Fayman et al. [3]	2017	23	Muscle-sparing mastectomy, VAC and skin grafting for mastectomy wound.
Konik et al. [1]	2017	53	Partial mastectomy and local tissue rearrangement.
Ward et al. [4]	2017	53	Radical mastectomy.
Lee et al. [8]	2016	31	Debridement and skin graft.
Pek et al. [9]	2015	27	Debridement and skin graft.
Lee et al. [6]	2015	31	Debridement and secondary wound closure using VAC.
Yang et al. [10]	2015	30	Debridement with conservation of the nipple and skin graft.
Yaji et al. [2]	2014	55	Wide debridement.
Pote et al. [11]	2013	22	Debridement and skin graft.
Vishwanath et al. [12]	2011	20	Mastectomy and skin graft.
Soliman et al. [13]	2011	61	Debridement with conservation of the nipple and skin graft
Keune et al. [14]	2009	47	Mastectomy.
Flandrin et al. [7]	2009	50	Debridement with conservation of the nipple, VAC and skin graft.
Venkatramani et al. [15]	2009	40	Mastectomy
Wong and Tan [16]	2008	38	Quadrantectomy and secondary wound closure.
Nizami et al. [17]	2006	54	Mastectomy and skin graft.
Rajakannu et al. [18]	2006	50	Mastectomy and skin grafting.
Shah et al. [5]	1999	50	Mastectomy.
